# Soft Palate and Uvula Thermal Burn Injury in an Adolescent Following Recreational Inhalation of Nitrous Oxide

**DOI:** 10.7759/cureus.97445

**Published:** 2025-11-21

**Authors:** Thomas Yeung

**Affiliations:** 1 Surgery, Great Western Hospital, Swindon, GBR

**Keywords:** airways, frostbite, inhalation, nitrous oxide, palate, thermal

## Abstract

Thermal injury refers to trauma to tissues as a result of exposure to a source of sufficiently high or low temperature. This mechanism of trauma may arise from direct contact with a heat source, e.g., scalding of skin secondary to immersion in hot water or following exposure to a stimulus below freezing temperature. Gases stored under pressure in a liquid state undergo adiabatic expansion upon decompression, which results in a rapid and significant decrease in temperature. Nitrous oxide is an example of a gas typically stored as a liquid in canisters prior to use. Tissue exposed to decompressed nitrous oxide undergoing adiabatic cooling can experience significant thermal injury, resulting in inflammation and, in severe cases, necrosis.

A 16-year-old girl presented to the Pediatric Emergency Department (ED) with marked dysphagia, odynophagia, and dysphonia, three days following recreational inhalation of nitrous oxide (N_2_O) gas from a canister. The patient reported on assessment that she had recreationally inhaled directly from a canister of compressed N_2_O during a recent party. She experienced diffuse throat pain following one breath and immediately stopped. Worsening dysphagia and dysphonia persisted over the following three days, and she attended the pediatric ED with her mother. The patient was otherwise healthy with no relevant medical history. Examination demonstrated a stable airway with ulceration and slough tissue at the soft palate and base of the uvula, secondary to thermal burn injury. She was apyrexic and observations were reassuring. Blood tests revealed a mildly elevated C-reactive protein (CRP) of 21 mg/L with otherwise unremarkable markers. A decision was made to admit under the Ear, Nose, and Throat (ENT) team. Regular intravenous dexamethasone, co-amoxiclav, and analgesia were subsequently initiated.

Following an expeditious recovery, the patient had improved quality of voice, tolerated a soft diet, and was discharged with a course of antibiotics and betamethasone mouthwash. Nitrous oxide, abused recreationally for its euphoric effects, is one of the most widespread illicit substances in the United Kingdom. While systemic sequelae, such as N_2_O-induced subacute spinal cord degeneration, are well-documented, there are few reports in the literature of localised thermal trauma to airway structures upon inhalation. This case aims to raise awareness of such complications, especially in adolescents, and to discuss ENT approaches to management.

## Introduction

Nitrous oxide (N2O), colloquially known as laughing gas, is an inhaled gas commonly utilised in medicine and dentistry. Its rapid onset and offset, in combination with mild sedative properties, contribute toward its role as a procedural anaesthetic agent, with applications ranging from fracture reduction to obstetric labour. Beyond the clinical context, nitrous oxide is abused recreationally for its euphoric effect. With approximately 230,000 individuals aged 16 to 24 years in England and Wales having inhaled nitrous oxide in 2021-2022 [1], it remains among the most widespread illicit substances in the United Kingdom.

While the systemic and neurological sequelae of nitrous oxide use are well-established, there are few reports in the literature of localised thermal injury to oropharyngeal structures upon inhalation. A search of the literature identified two recent similar cases in the West of thermal burn to the palate and buccal mucosa arising from nitrous oxide inhalation [2-3]. The first of the two cases describes a similar mechanism of injury, while the second highlights a more severe injury with significant airways oedema requiring endotracheal intubation. This unusual case of localised frostbite injury secondary to nitrous oxide inhalation aims to raise awareness of such complications, especially in adolescents, and to discuss ENT approaches to management.

Thermal injury in cases of nitrous oxide inhalation occurs as a result of exposure to very low temperatures upon decompression of liquid nitrous oxide into the gaseous state. According to the thermodynamic principle of adiabatic expansion, most gases cool upon expansion, where no external heat is applied [4]. Decompressed nitrous oxide can reach temperatures of minus 40 degrees Celsius, resulting in significant injury, as a result of thawing and freezing following vasoconstriction and vasodilation in affected tissue. The formation of ice crystals within tissue, which causes injury to cellular membranes, is a further mechanism of trauma [5]. Such injuries can result in tissue ischaemia and loss, resulting in pain, infection and scarring. Mild cases may be conservatively managed with antibiotics and wound care, while large areas of tissue loss, especially of skin and soft tissue, may necessitate surgical debridement and reconstruction [6].

## Case presentation

A 16-year-old girl, accompanied by her mother, presented to the paediatric Emergency Department (ED) three days following a party, with severe odynophagia, dysphagia and dysphonia. On further questioning, the patient revealed that she had recreationally inhaled nitrous oxide directly from a compressed canister. Immediately following one breath, she experienced diffuse, severe throat pain and stopped as a result. Odynophagia continued into the following morning, accompanied by dyspnoea at rest and minimal oral intake; tolerating only small sips of fluid. The mother also reported that her voice had become increasingly hoarse. A review of systems revealed no other remarkable features. She attended the ED as a result of worsening symptoms and parental concern.

**Figure 1:**
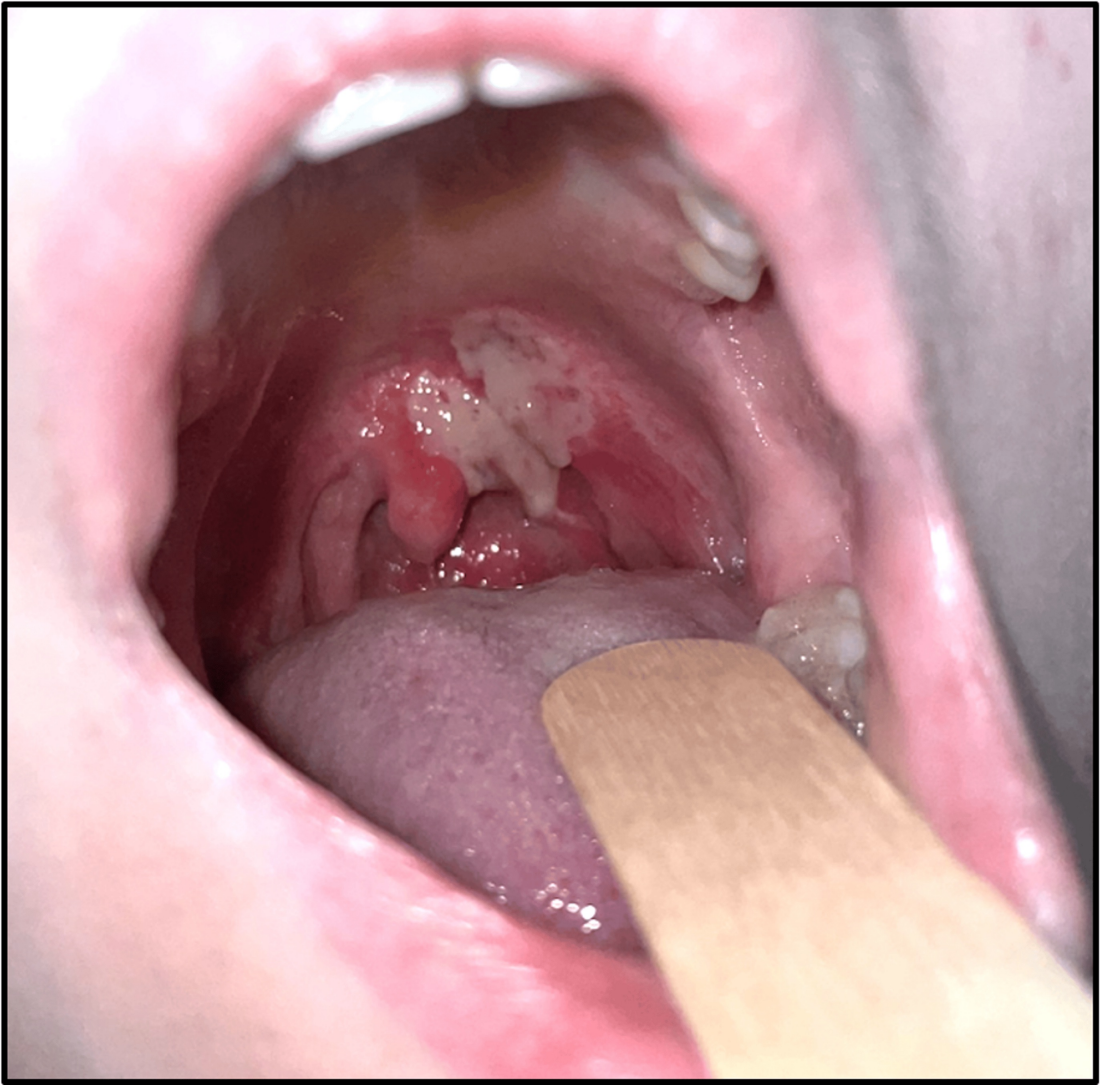
Patchy slough with surrounding erythema at the soft palate, base of uvula and posterior pharyngeal wall

She was otherwise fit and well with no relevant medical or ENT history. She took no regular medications, denied smoking and had no allergies. There were no safeguarding concerns on assessment. Observations were within normal range with a Paediatric Early Warning Score (PEWS) of zero and oxygen saturations of 100% on room air. There was no evidence of airway compromise, and the patient was not stridulous nor in respiratory distress. Inspection of the oral cavity revealed a patch of sloughy tissue to the left soft palate and uvula base, measuring roughly 2 x 2 cm, with surrounding erythema extending to the posterior pharyngeal wall (Figure 1). There was no mucosal bleeding and no purulent discharge. The tongue was healthy, and there was no other significant oropharyngeal swelling. Examination of the neck did not demonstrate any significant tenderness or lymphadenopathy; there was no trismus, and the patient had a satisfactory range of neck movement. The patient was unwilling to undergo flexible nasendoscopy (FNE) to assess for laryngeal oedema.

Blood tests on admission revealed a mildly elevated C-reactive protein (CRP) of 21 mg/L, with unremarkable white cell and neutrophil counts. A venous blood gas demonstrated normal acid-base balance and a lactate of 0.6. mmol/L. See Table 1.

**Table 1:** Blood test results on admission

Test	Result	Reference range	Units
C-reactive protein	21	0.0 - 5.0	mg/L
Lactate	0.6	0.5 - 2.2	mmol/L

A decision was made to admit to the paediatric ward under joint care with ENT, for observation of the airway. Pharmacological management involved a stat dose of dexamethasone and antibiotic cover with regular intravenous co-amoxiclav. Analgesia included paracetamol, ibuprofen, topical benzydamine spray and oral morphine solution. Radiological imaging was not indicated. The patient responded well to conservative management, remaining clinically stable overnight, with reduced pain and improving dysphonia the following day. A trial of a soft diet was started, and the patient demonstrated good improvement in oral intake. She was discharged home with safety-netting advice and a further five-day course of oral co-amoxiclav, a week of betamethasone mouthwash and benzydamine spray. A face-to-face clinic follow-up was arranged for six weeks post-discharge; however, the patient did not attend.

## Discussion

Multiple neurological and psychiatric systemic effects of nitrous oxide abuse are well-documented in the literature. Repeated exposure is associated with subacute degeneration of the spinal cord, which can present as paraesthesia, motor deficit and ataxia [7]. Interference in the Vitamin B12 synthesis pathway, resulting in deficiency, is the responsible pathophysiology [8]. A review of psychiatric sequelae highlights a plethora of manifestations, including hallucinations, cognitive impairment and mood disorder [9]. A case of psychomotor disturbance accompanied by psychotic features involving persecutory delusions and partial insight loss has been described in a 19-year-old chronic abuser of nitrous oxide [10].

Decompression of a pressurised liquid, i.e., deodorant or nitrous oxide, into the gaseous state leads to a rapid decrease in temperature. While frostbite injury to the oropharynx and upper airways secondary to nitrous oxide is less frequently described, this has the potential to confer significant morbidity and mortality, as with thermal airway injury of any other mechanism. Resultant glottic and supraglottic oedema may result in acute airway compromise in patients in extremis, requiring endotracheal intubation, and in cases of failed intubation, surgical tracheostomy or emergency front-of-neck access [11].

In this case report, frostbite injury to the soft palate and uvula was mild, likely resulting from the very short duration of exposure. Exposure time and severity of airways injury are positively correlated in smoke inhalation [12], although it should be noted that the mechanism in such cases conversely is high-temperature thermal burn. There was an index of suspicion for laryngeal oedema secondary to the patient’s acute dysphonia; however, the resolution of this during her admission was reassuring. Fiberoptic nasoendoscopy (FNE) evaluation, if allowed by the patient, would have assisted in excluding epiglottic or supraglottic swelling [13]. Prudent observation, especially in the absence of an FNE, was necessary to monitor for deterioration at an early stage. The planned follow-up at six weeks would have allowed for re-examination of the oral cavity; however, the patient did not attend the clinic.

## Conclusions

This case report demonstrates a case of thermal injury to the soft palate and uvula in a paediatric patient, conservatively managed with intravenous steroids, antibiotics and adequate analgesia. While this patient did not suffer acute airway compromise, the unusual mechanism of trauma to the throat secondary to nitrous oxide inhalation remains underreported in the literature. The potential for significant morbidity as a result of thermal injury to the airways, especially adolescents who may represent a population more prone to substance misuse, must be well-understood by paediatricians, anaesthetists and ENT clinicians who may encounter and manage similar presentations while on call.
